# Single Cell Gene Profiling Revealed Heterogeneity of Paracrine Effects of Bone Marrow Cells in Mouse Infarcted Hearts

**DOI:** 10.1371/journal.pone.0068270

**Published:** 2013-07-05

**Authors:** Yanhua Li, Xinhong Guo, Qiao Xue, Mei Zhu, Lei Gao, Yu Wang

**Affiliations:** Institute of Geriatric Cardiology, the General Hospital of Chinese People’s Liberation Army (PLA), Beijing, China; Northwestern University, United States of America

## Abstract

It is now recognized that transplantation of bone marrow cells (BMCs) into infarcted hearts has the capacity to improve the cardiac function through paracrine effects. However, detailed expression levels of paracrine factors in BMCs in infarcted hearts are poorly described. By use of laser capture microdissection combined with real-time PCR, we depicted the expression profiles of paracrine factors in infarcted hearts versus normal hearts. Consistent with the *in vivo* observation, a similar expression pattern was evidenced in cultured BMCs. Furthermore, BMCs displayed heterogeneity of paracrine effects in infarcted hearts as analyzed at the single cell level using single cell PCR. Interestingly, the CD45^+^ subpopulation showed higher expression levels of angiogenic factors compared to other subpopulations. Finally, most angiogenic factors were induced under the microenvironment of infarction. Our study demonstrated the heterogeneity of paracrine effects in BMCs at single cell level in infarcted hearts, highlighting preferential expression of angiogenic factors in the CD45^+^ subpopulation. These findings broaden our understanding of paracrine effects of BMCs *in vivo*, and offer new insights into BMCs therapy in myocardial infarction (MI).

## Introduction

Despite advances in our understanding and treatment of coronary artery disease, myocardial infarction (MI) remains a major cause of morbidity and mortality worldwide. Recently, stem cell therapy is recognized as a promising therapeutic strategy in the protection and repair of damaged myocardium after infarction [1], [2], [3]. Growing experimental studies and clinical trials have been carried out using BMCs, displaying encouraging outcomes in the improvement of cardiac function, which offered the fascinating possibility of the application of BMC-based therapy in MI [4], [5], [6], [7], [8], [9].

In order to ensure the safety and effectiveness of BMC therapy, extensive studies were performed to dissect the mechanisms underlying their therapeutic actions. Transdifferentiation into cardiomyocytes and vascular lineage cells has been originally proposed as the principal mechanism accounting for improved cardiac function. Anversa and colleagues illustrated that Lin-c-Kit^+^ (Hematopoietic stem cells enriched) cells from BMCs can give rise to cardiac cells, and regenerate 68% of the infarcted area with novel cardiomyocytes [5]. Conversely, however, other groups failed to detect cardiac transdifferentiation in hearts transplanted with BMCs derived hematopoietic stem cells (HSCs) [10], [11]. Given the ongoing scientific debates, a different hypothesis has been raised, emphasizing paracrine effects of stem cells in the treatment of MI [12]. For example, Takahashi et al. injected conditioned medium from bone marrow derived mononuclear cells (BM-MNCs) into infarcted hearts and observed an overall improvement of cardiac function [13]. Simultaneously, a considerable number of studies focused on the regulation of paracrine effects in cultured BMCs [14], [15], [16]. However, the paracrine effects of BMCs *in vivo*, as well as responses of paracrine actions challenged by infarction, are not fully understood. In addition, given that BMCs are composed of different cell populations, analyses of the paracrine effects that distinguish the different subpopulations were rarely reported.

In this study, we depicted paracrine effects of BMCs *in vivo*, with particular focus on the comparison between infarcted and normal hearts. Moreover, we uncovered the heterogeneity of paracrine effects of BMCs in infarcted hearts using single cell PCR, and revealed that the CD45^+^ subpopulation in BMCs preferentially expressed angiogenic factors.

## Materials and Methods

### Ethics Statement

All animal studies have been approved by the Animal Care and Use Committee of the General Hospital of Chinese PLA.

### Animals

Myocardial infarction (MI) surgery in female SCID mice, 12 weeks old, was used in this study. Male green fluorescent protein (GFP) transgenic mice of FVB background, 6–8 week old, were used as donors for bone marrow cells (BMCs) isolation. All transgenic mice were purchased from The Laboratory Animal Center of Academy of Military Medical Sciences.

### Myocardial Infarction and Stem Cells Transplantation

Myocardial infarction was created in female SCID mice by permanent ligation of left anterior descending coronary artery (LAD). The animals were anesthetized with sodium pentobarbital (50 mg/kg IP) and mechanically ventilated. After a left-sided minithoracotomy, the heart was exposed and LAD was ligated by 7-0 ethicon suture at just below the atrioventricular border. The mice were then randomly given an intraventricular injection with 31-gauge needle of one of the following: PBS (20 µl), BMCs (1×10^6^, 20 µl), CD45^−^ BMCs (5×10^4^, 20 µl) or CD45^+^ BMCs (5×10^4^, 20 µl).

### Laser Capture Microdissection (LCM)

Murine hearts were removed 5 days post surgery, embedded in optimal cutting temperature (OCT), and then frozen by liquid nitrogen. Briefly, cyrosections (7 µm thickness, 350 µm apart) of left ventricle were prepared on polyethylene naphthslate membrane-coated slides (MicroDissect GmbH). GFP positive cells were captured by laser microdissection (Leica LCM system). The captured cells were subjected to next step of experiments.

### Echocardiography Study

For echocardiography study, mice were anesthetized by 2% isoflurane with oxygen (0.8 L/min). Mouse echocardiography was conducted using an echocardiograph (Philips) pre, 1, 4 and 10 days after surgery as previously described. Fractional shortening (FS) were employed to evaluate heart function of mouse.

### Immnohistochemistry and Immunoflurescence Staining

Mouse hearts were harvested 5 days after surgery and subjected to frozen sections (7 µm thickness, 350 µm apart). The sections were subjected to hematoxylin and eosin staining (H&E staining), or incubated with rabbit anti-CD31 (Abcam) antibody at 4°C overnight, followed by secondary antibodies against rabbit (Invitrogen) at 37°C for 1 hour. Confocal microscopy was used to observe the specific staining.

### Real-time Quantitative PCR Analysis

Total RNA was extracted from tissues or cultured cells, and 0.5 µg RNA was used in the following reverse transcription–polymerase chain reaction. The obtained cDNA were subjected to real-time PCR (Applied Biosystems; ABI) to evaluate the expression level of various paracrine factors. All probes used in our study were purchased from ABI.

### Isolation and Culture of BMCs

In brief, mice femurs were removed immediately after sacrifice, and flushed by phosphate saline buffer (PBS) with 2% fetal bovine serum (FBS). The obtained cells were lysed by ammonium-chloride-potassium (ACK) lysing buffer (Gibco) to eliminate red blood cell contamination. The resulting cells were either cultured in BMC culture medium (STEMCELL Technologies) or subjected to isolation of CD45^+^ cells. Magnetic activated cell sorting (MACS, Miltenyi) was employed to collect CD45^+^ cells from whole BMCs. The cultured BMCs were exposed to either hypoxia (1% O_2_, 5% CO_2_, 94% N_2_) or normoxia condition for 48 hours. After that, the total RNA was extracted for evaluation of various paracrine factors.

### Fluorescence Activated Cell Sorting (FACS) and Single Cell Isolation

SCID mice were sacrificed 5 days after surgery. Hearts were removed and chopped up in microfuge tubes as fine as possible, then small pieces were moved to 10 cm culture dishes followed by collagenase typeI (1 mg/ml, Sigma) digestion for 1 hour at 37°C. Afterwards, cells were spun down to form a pellet, which was further digested with 0.25% trypsin at 37°C for another 5–10 min. The resulting cells were subjected to FACS to sort GFP^+^ cells.

### High through-put Single-Cell qPCR Analysis

Inventoried TaqMan assays (20×, Applied Biosystems) were pooled and diluted to a final concentration of 0.2× for each of the 48 probes. Individual cells were collecteddirectly into 10 µl RT-PreAmp Master Mix (5.0 µl CellsDirect 2× Reaction Mix (Invitrogen); 2.5 µl 0.2× assay pool; 0.5 µl RT/Taq enzyme (CellsDirect qRT-PCR kit, Invitrogen); 2.0 µl TE buffer). After that, the mixture of single cell was applied to sequence specific reverse transcription at 50°C for 20 min. Subsequently, cDNA was used as a template for partial sequence-specific amplification by following protocol: denaturing at 95°C for 15 s, and annealing and amplification at 60°C for 4 min for 18 cycles. These preamplified products were analyzed with Universal PCR Master Mix and inventoried TaqMan gene expression assays (ABI) in 48.48 Dynamic Arrays on a BioMark System (Fluidigm). Ct values were calculated from the system’s software (BioMark Real-time PCR Analysis; Fluidigm).

### Single-Cell Data Processing

All Ct values of genes were converted into relative expression levels by subtracting the values from the assumed baseline value of 32. The resulting values were then normalized to the endogenous controls by subtracting the average of its β-actin and GAPDH expression levels.

### Statistical Analysis

Data are expressed as mean±standard error of the mean (SEM). Statistical analysis involved use of the Student’s *t*-test for comparison of two groups. A *P*<0.05 was considered statistically significant.

## Results

### Gene Expression Profiling of Paracrine Effects of BMCs in Normal Versus Infarcted Hearts

**Figure 1:**
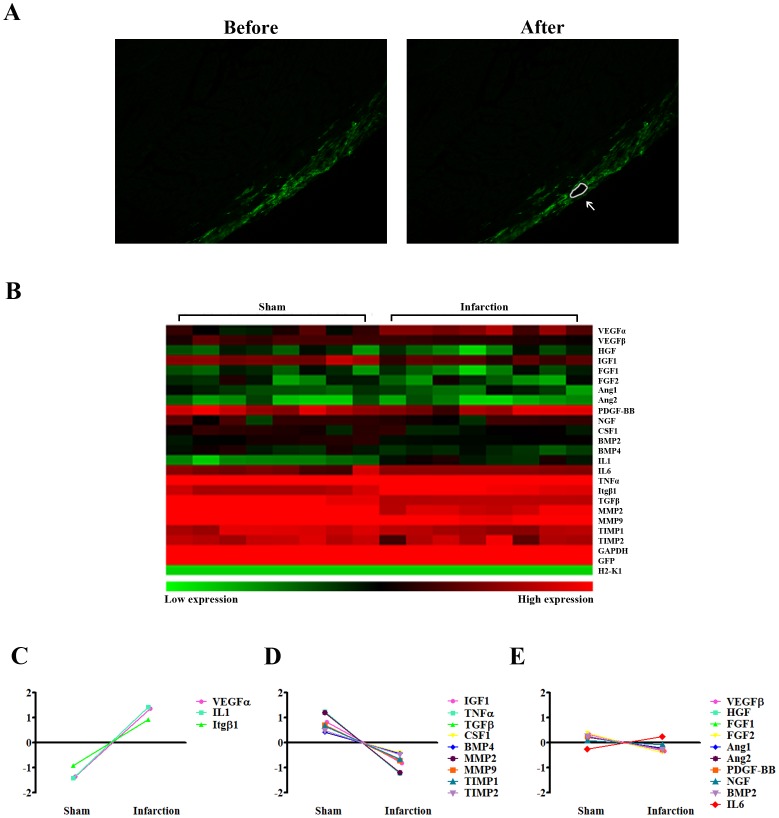
Gene expression profiling of paracrine effects of BMCs in normal versus infarcted hearts. (A) Representative pictures of LCM performed in frozen slides of normal and infarcted hearts. Removed GFP+ cells (white arrow) were indicated by white lines. (magnification 100×). (B) An array displayed overall expression profile of paracrine factors synthesized by BMCs in normal and infarcted hearts 5 days post surgery. (n = 8 per group). (C–E) Quantitative analysis of gene expression in normal and infarcted hearts. Values are mean of 8 mice per each group.

Using a LAD ligation model, we first examined gene expression levels of different paracrine factors of BMCs *in vivo*. 5 days after surgery, injected cells were evidenced by GFP signal. To assess their paracrine effects in hearts, GFP^+^ cells (200 cells) were captured using LCM in both sham and infarcted hearts (Figure 1A). The expression profile of paracrine effects of the injected cells was evaluated by real-time PCR array. When the cells were analyzed as a single population, they displayed similar expression patterns in terms of gene expression of paracrine factors (Figure 1B). Among those factors, VEGFα and IL1 were significantly increased in infarcted hearts (Figure 1C). Conversely, IGF1, TNFα, TGFβ, CSF1, BMP4, MMP2, MMP9, TIMP1 and TIMP2 were markedly decreased (Figure 1E). No significant changes were detected regarding the expression of VEGFβ, HGF, FGF1, FGF2, Angiogenin1 (Ang1), Angiogenin2 (Ang2), PDGF-BB, NGF, BMP2 or IL6 (Figure 1D). The above results suggested that BMCs failed to enhance most of the paracrine factors under hypoxia challenge *in vivo*.

### Heart Function was Improved by BMCs Injection

**Figure 2:**
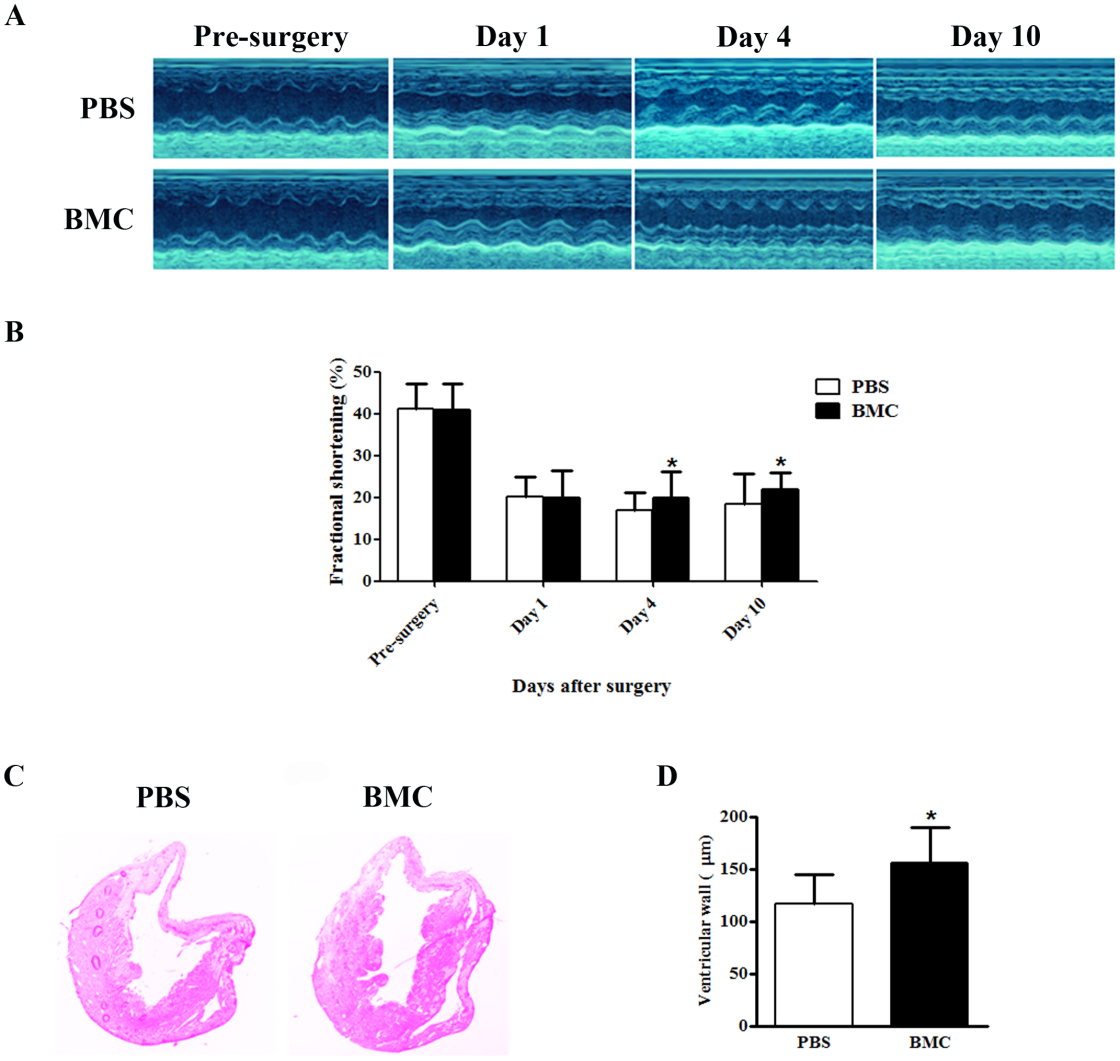
Heart Function Was Improved by BMCs Injection. (A) Representative images of M-mode echocardiogram of infarcted hearts with PBS or BMCs injection at pre-surgery, day 1, 4 or 10. (B) Quantification of fractional shortening (%) at different time points in both groups. *P<0.05 versus PBS injection group, n = 8. (C) Representative images of HE staining 5 days after surgery from both groups. Magnification is 100×. (D) Quantification on histological sections from PBS or BMCs injected hearts. Values are means±SEM. n = 8 per group. *P<0.05 versus PBS.

To assess cardiac function after BMCs transplantation, echocardiograms were applied in our study at pre-surgery, 1, 4, and 10 days post-surgery (Figure 2A & B). At day 1 post-surgery, both BMCs injected group and PBS group displayed significant reduction in cardiac function, characterized by decreased fractional shortening (FS), which dropped from 41% to 20%, suggesting successful MI surgery. Intriguingly, compared to PBS injected group, BMCs injected group displayed moderate but statistically significant increase in FS at 4 days (16.97% vs. 20%) and 10 days (18.35% vs. 21.88%) after surgery, respectively. To further confirm the effect of BMCs injection, HE staining was employed in our experiments to determine the ventricular wall thickness. At day 10, ventricular wall thickness was increased in BMCs injected group compared to PBS group (155.6±67.6 vs. 117.0±55.8 µm, n = 8 mice for each group, P<0.05; Figure 2C & D). These results suggested that BMCs injection was capable of improving cardiac function after infarction.

### Cultured Bone Marrow Cells Displayed Similar Expression Pattern of Paracrine Factors in Response to Hypoxia Stimulation

**Figure 3:**
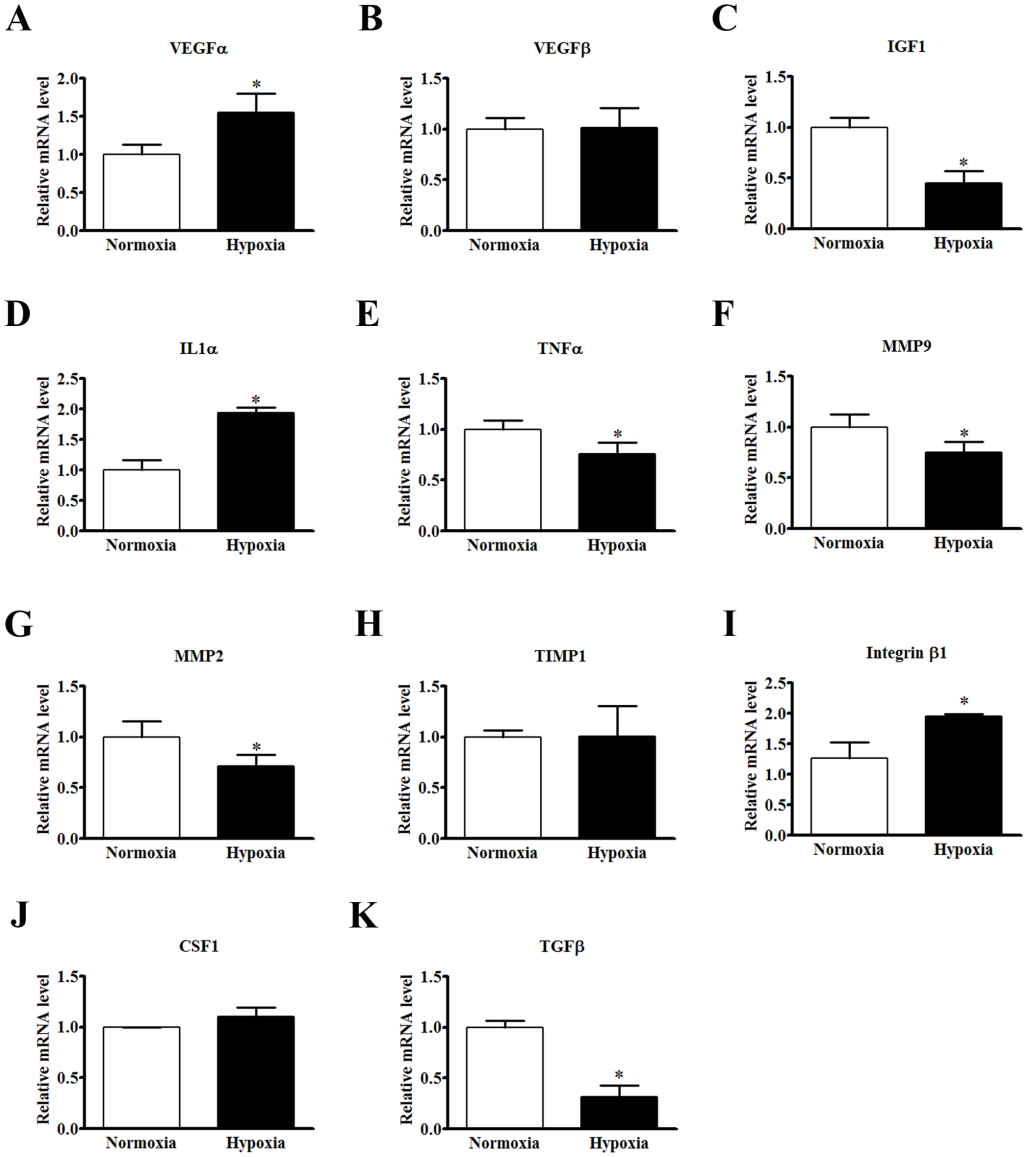
Paracrine effects of cultured BMCs in response to hypoxia. (A–K) Quantification of relative mRNA level of paracrine factors in cultured BMCs 48 hours after hypoxia stimulation. *P<0.05 versus normoxia condition.

*In vitro* experiments were utilized to further confirm the paracrine response of BMCs induced by hypoxia. Consistent with the observation *in vivo*, the expression of VEGFα and IL1α was induced by 48-hour hypoxia stimulation (1.55- and 1.94-fold increase, respectively. Figure 3A, D). In contrast, the expression of IGF1, TNFα, MMP2, MMP9 and TGFβ was inhibited under hypoxia condition (decreased by 45.3%, 75.7%, 71.5%, 74.9% and 31.5%, respectively. Figure 3C, E, F, G, K). However, there were no significant changes in the expression of VEGFβ, TIMP1 or CSF1 (Figure 3B, H, J).

### BMCs Displayed Heterogeneity Regarding Paracrine Effects in Infarcted Hearts

**Figure 4:**
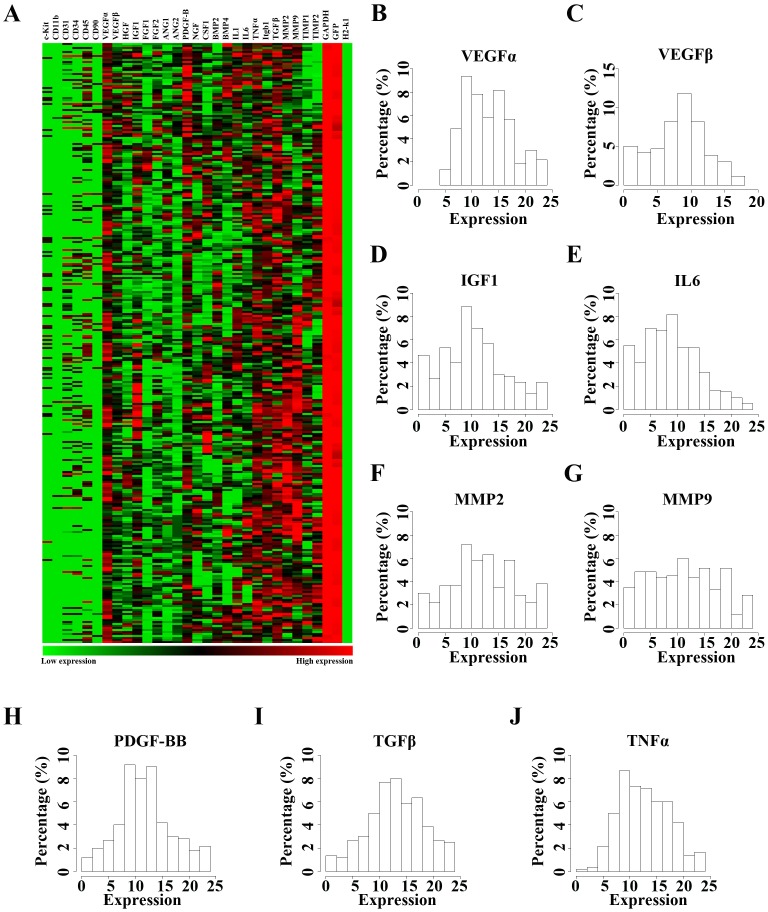
BMCs displayed heterogeneity regarding paracrine effects in infarcted hearts. (A) A heat map showing that gene expression levels of different paracrine factors from 300 individual BMCs collected from infarcted hearts. (B–J) Population distribution plots (horizontal axis represents expression level; vertical axis represents percentage of total cell population) uncovered that single BMCs collected from infarcted hearts possessed considerable heterogeneity with respect to synthesis of paracrine factors.

Since BMCs comprise different subpopulations of cells, and thus display multilineage differentiation potential, we then assessed the heterogeneity of BMCs with regard to paracrine effects. Transplanted GFP^+^ BMCs were collected 5 days after infarction surgery by FACS, followed by analysis of paracrine effects at single cell level (Figure S1B). To exclude local cell contamination and cell fusion, we applied probes against SRY (data not shown), GFP and H2-K1 in following single cell PCR (Figure S1A). A total of 300 transplanted BMCs were harvested from 6 infarcted hearts (50 cells per mouse), and analyzed by single cell real-time PCR array with a total of 31 specific probes. Compared to analysis as a single population, the transplanted cells exhibited heterogeneity of paracrine effects at single cell level (Figure 4A). In addition, the frequency distribution was performed to evaluate the heterogeneity of paracrine effects in single cells, which was evidenced by the horizontal spread of a histogram plot (Figure 4B–J). The expression of most of major paracrine factors, including VEGFα, VEGFβ, IGF1, IL6, MMP2, MMP9, PDGF-BB, TGFβ and TNFα, which are involved in the pathophysiological process after MI, showed variation among different cells.

### Various Angiogenic Factors were Preferentially Highly Expressed in CD45^+^ BMCs in Infarcted Hearts

**Figure 5:**
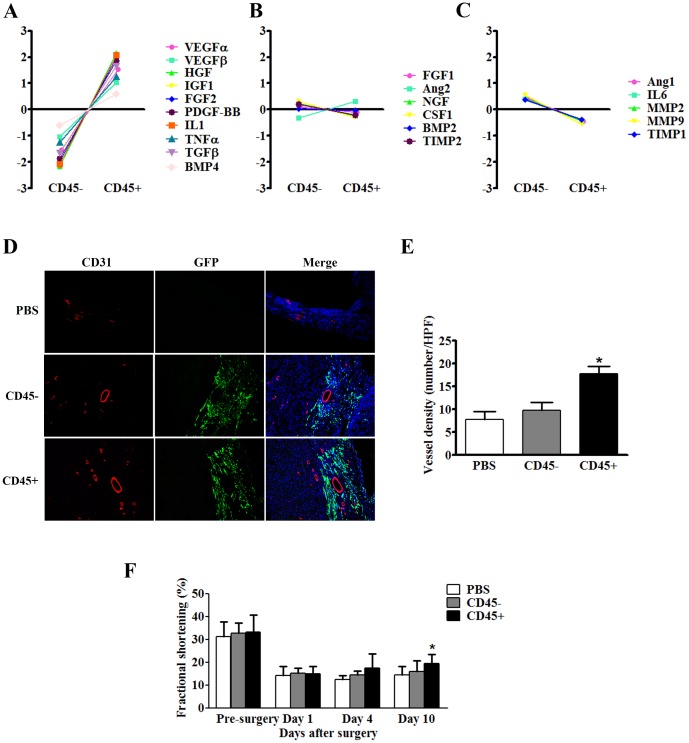
Expression profile of paracrine factors in CD45^+^ subpopulation versus CD45^−^ subpopulation of BMCs in infarcted hearts. (A) The expression levels of most paracrine factors were significantly higher in CD45^+^ cells compared to that in CD45^−^ cells. (B) There was no significant difference of expression levels of 6 factors. (C) Compared to CD45^−^ cells, only 5 factors expression levels were lower in CD45^+^ cells. (D) Representative images of immunostaining against CD31 (red fluorescence) in infarcted hearts in each group. Green indicated injected cells. Magnification is 100×. (E) Quantification of vessel density in each group, The average number of vessels from 8 randomly chosen high-power fields (100×) was counted from each mouse. (n = 8, *P<0.05 versus CD45- BMCs group). (F) Quantification of fractional shortening (%) at different time points in each group. *P<0.05 versus CD45^−^ BMCs injection group, n = 8.

Due to the diversity of components, as well as the heterogeneity of paracrine effects in BMCs, different markers of subpopulations, such as CD31, CD34, CD45 and CD90, were also employed in our single cell PCR study to compare the differentially expressed genes among different subpopulations (Figure 4A). Compared to other subpopulations, the CD45^+^ subpopulation was intriguing in that it highly expressed angiogenic factors. 5 days after MI and BMCs transplantation, various factors implicated in angiogenesis, including VEGFα, VEGFβ, HGF, IGF1, FGF2, PDGF-BB, IL1, TNFα, TGFβ and BMP4, were highly expressed in CD45^+^ cells relative to CD45^−^ cells (Figure 5A). Interestingly, some of the factors were involved in the degradation and remodeling of extracellular matrix (ECM), such as MMP2, MMP9 and TIMP1, were highly expressed in CD45^−^ cells (Figure 5C). However, no significant differences in the expression levels of FGF1, Ang2, NGF, CSF1, BMP2 and TIMP2 were observed between these two populations (Figure 5B). Collectively, these data suggested that the CD45^+^ subpopulation plays a critical role in angiogenesis after MI.

### CD45^+^ BMCs Enhanced Angiogenesis and Cardiac Function after Infarction

In order to examine angiogenic effect of CD45^+^ BMCs *in vivo*, myocardial infarction surgery was performed followed by injection of PBS, CD45^−^ BMCs (5×10^4^) or CD45^+^ BMCs (5×10^4^), respectively. Five days after surgery, CD45^+^ BMCs injection group showed higher density of CD31 positive staining compared to PBS and CD45^−^ BMCs groups, revealing that CD45^+^ BMCs possessed higher angiogenic effect *per se* (Figure 5D & E). In agreement with this angiogenic effect, cardiac function was improved in CD45^+^ BMCs group compared to PBS and CD45^−^ BMCs groups at day 4 (17.5% vs. 12.5% and 14.4%) and day 10 (19.4% vs. 14.3% and 15.9%), respectively (Figure 5F).

### The Expression of Angiogenic Factors were Induced in CD45^+^ Cells in Infarcted Hearts

**Figure 6:**
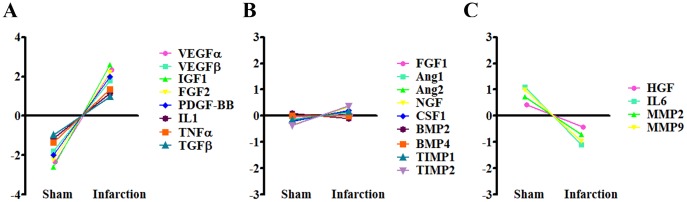
The expression levels of paracrine factors of bone marrow derived CD45^+^ cells in infarcted hearts versus normal hearts. (A) Considerable amount of paracrine factors of CD45^+^ cells were upregulated in infarcted hearts. (B) Factors without significant changes. (C) 4 factors were downregulated in response to myocardial infarction. (n = 8 per each group).

Based on the above results, we asked how angiogenic factors are regulated in response to hypoxia condition *in vivo*. After isolation of CD45^+^ BMCs *in vitro*, 5×10^4^ cells were immediately injected into hearts with (MI) or without myocardial infarction (Sham). The transplanted cells were collected by LCM 5 days after surgery. Real-time PCR revealed that the expression of most of angiogenic factors, including VEGFα, VEGFβ, IGF1, FGF2, PDGF-BB, IL1, TNFα and TGFβ, were enhanced in infarcted hearts compared to sham group (Figure 6A). By contrast, the expression of a few factors was inhibited in infarcted hearts (Figure 6C). There were no significant changes in the expression of FGF1, Ang1, Ang2, NGF, CSF1, BMP2, BMP4, TIMP1 and TIMP2 (Figure 6B). Taken together, these results revealed that, compared to CD45^−^ cells, CD45^+^ BMCs were more responsive to hypoxia stimulation *in vivo* with regard to the secretion of angiogenic factors, which may promote angiogenesis and myocardial recovery after MI.

## Discussion

Clinical trials have been carried out worldwide on cell-based therapy, with particular interest in the delivery of autologous cells derived from bone marrow to treat ischemic heart diseases [17], [18]. In order to obtain safe and effective therapeutic effects, considerable studies were performed to investigate the underlying mechanisms of BMC therapy in ischemic hearts. Rather than direct cell transdifferention, there is a growing body of evidence supporting the hypothesis that paracrine mechanisms mediated by factors secreted from BMCs play a crucial role in the protection and recovery of damaged myocardium. However, due to limited approaches for assessing paracrine effects *in vivo*, there was lack of proof of how BMCs secret paracrine factors in hearts, especially under the challenge of ischemia. Our study, for the first time to our knowledge, delineated the regulation of paracrine factors released by BMCs *in vivo*, uncovered the heterogeneity of paracrine effects of BMCs at a single cell level in infarcted hearts, and revealed preferential secretion of angiogenic factors in the CD45^+^ subpopulation in ischemic myocardium. Although some positive outcomes of paracrine effect-based BMCs therapy, such as improved capillary density and cardiac function [8], [19], [20], [21], have been reported in human clinical trials and experimental studies, the precise expression levels of paracrine factors in BMCs in infarcted hearts remain unclear. By use of GFP transgenic mice and LCM, we had the unique opportunity to monitor the expression of various paracrine factors *in vivo*, and showed that BMCs expressed a broad spectrum of paracrine factors in infarcted hearts. Given the hypoxic microenviroment in infarcted hearts, it is important to study the response of BMCs challenged by hypoxia. Many studies elucidated the regulation of gene expression of paracrine factors in BMCs under hypoxia stimulation *in vitro*, as well as enhanced level of paracrine factors in infarcted hearts treated with BMCs transplantation [15], [22], [23]. However, few studies investigated the response of BMCs in infarcted hearts compared to normal hearts. Surprisingly, in our study, we found that only a few paracrine factors were upregulated in infarcted hearts, whereas most of the paracrine factors were downregulated or not affected by hypoxia stimulation. Our results were not controversial with other groups showing increased VEGF, bFGF, HGF, IGF1 and adrenomedullin in injured hearts treated with BMCs [22], because their results were based on the comparison between cell transplantation and non-transplantation groups, rather than cell transplantation in infarcted hearts versus normal hearts.To confirm the observations *in vitro*, we studied the regulation in cultured cells, where only a few paracrine factors were induced upon hypoxia stimulation, which was consistent with results from other groups, as well as with our *in vivo* data.Since bone marrow cells exemplify a typical adult stem cell source containing different cell populations with diverse characteristics under various stimuli, studying the response to hypoxia of subpopulations in the heart after BMCs administration facilitates our understanding of the complex process of heart recovery, and helps optimize therapeutic strategies using different subpopulations. By application of single cell PCR, we uncovered the heterogeneity of BMCs in expression of paracrine factors in infarcted hearts. Based on the results, one would speculate that different subpopulations in BMCs possess different expression profiles of paracrine factors. Identification of firemen, bystanders or criminals in BMCs may facilitate the selection of specific subpopulations for different therapeutic purposes in MI.Using identified markers of different subpopulations in BMCs, we were able to analyze the characteristics of paracrine effects among different subpopulations. Our results revealed a preferential expression of angiogenic factors in CD45^+^ cells. The CD45 antigen is expressed on all cell surfaces of hematopoietic origin except for erythrocytes and platelets. Using CD45 as the marker, Balsam L. et al. reported that BMC-derived stem cells adopted mature haematopoietic [10]. However, since BMCs were capable of transdifferentiating into cells of diverse phenotypes in infarcted hearts, more specific markers should be applied in future studies to define the exact cell type of CD45^+^ cells. Besides higher expression levels in CD45^+^ cells, most angiogenic factors were also induced under hypoxia stimulation *in vivo*, indicating a desirable response of CD45^+^ cells in infarcted hearts. Due to the small number of CD45^+^ cells in transplantation, we were not able to compare the outcomes of stem cell transplantation between BMCs and CD45^+^ cells. Further studies need to be conducted to address the questions regarding heart protection, remodeling, repair, and regeneration using CD45^+^ cells.

In summary, our study demonstrated the heterogeneity of paracrine effects in BMCs, and revealed that the CD45^+^ subpopulation of BMCs have higher expression levels of angiogenic factors and a more desirable response under hypoxia challenge compared to CD45^−^ cells. Together, these findings broaden our understanding of stem cell therapy at the single cell level, and offer new insights into optimization of stem cell type selection for therapeutic applications.

## Supporting Information

Figure S1 **Strategy used in our study to isolate single injected cell without local cell contamination and cell fusion (A).** (B) Representative images of FACS in single cell collection. GFP cells were sorted and analyzed afterwards.
(TIF)
