# Supplementary figures and images for: Single Cell Gene Profiling Revealed Heterogeneity of Paracrine Effects of Bone Marrow Cells in Mouse Infarcted Hearts
Source: PLoS One. 2013 Jul 5;8(7):e68270. doi: 10.1371/journal.pone.0068270 (PMC3702556; doi:10.1371/journal.pone.0068270)

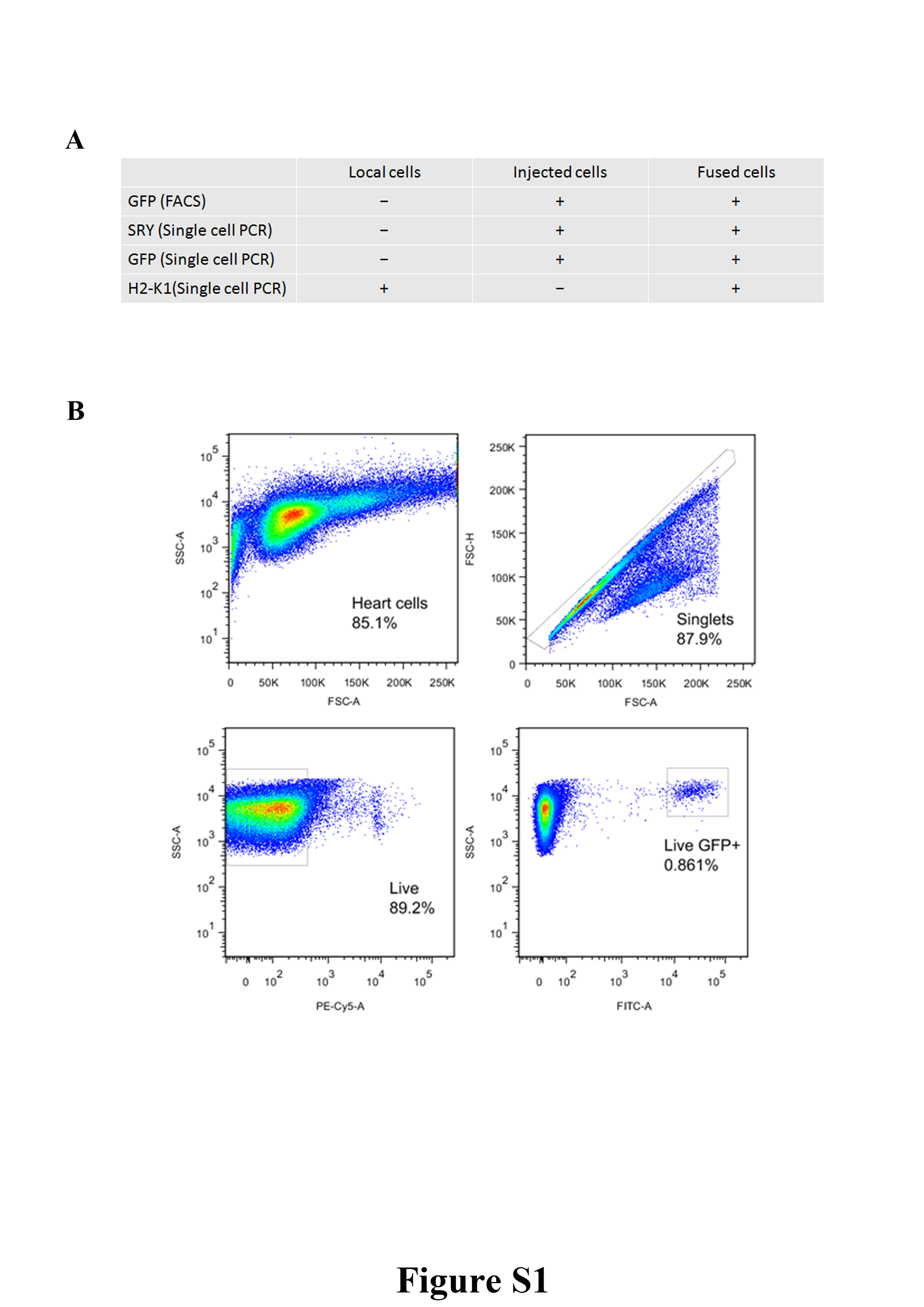

Supplement: Figure S1 — Strategy used in our study to isolate single injected cell without local cell contamination and cell fusion (A). (B) Representative images of FACS in single cell collection. GFP cells were sorted and analyzed afterwards. (TIF) [file pone.0068270.s001.tif]
